# How a dynamic optical system maintains image quality: Self-adjustment of the human eye

**DOI:** 10.1167/jov.21.3.6

**Published:** 2021-03-03

**Authors:** Agnieszka Jóźwik, Magdalena Asejczyk-Widlicka, Piotr Kurzynowski, Barbara Krystyna Pierscionek

**Affiliations:** 1 Department of Optics and Photonics, Faculty of Fundamental Problems of Technology, Wroclaw University of Science and Technology, Wroclaw, Poland; 2 Department of Optics and Photonics, Faculty of Fundamental Problems of Technology, Wroclaw University of Science and Technology, Wroclaw, Poland; 3 Department of Optics and Photonics, Faculty of Fundamental Problems of Technology, Wroclaw University of Science and Technology, Wroclaw, Poland; 4 School of Life Sciences and Education, Staffordshire University, Stoke-on-Trent, Staffordshire, UK

**Keywords:** optical self-adjustment, intraocular pressure, eye biometry, optical eye modelIntroduction

## Abstract

The eyeball is continually subjected to forces that cause alterations to its shape and dimensions, as well as to its optical components. Forces that induce accommodation result in an intentional change in focus; others, such as the effect of intraocular pressure fluctuations, are more subtle. Although the mechanical properties of the eyeball and its components permit mediation of such subtle forces, the concomitant optical changes are not detected by the visual system. Optical self-adjustment is postulated as the mechanism that maintains image quality. The purpose of this study was to investigate how self-adjustment occurs by using an optical model of the eyeball and to test the requisite optical and biometric conditions.

## Introduction

The material properties of the eyeball and its components are such as to allow sufficient flexibility to support the vital dynamics of physiological and optical processes. These include intraocular muscle action in accommodation, extraocular muscle forces in eye movement, and intraocular pressure (IOP) fluctuations, which can range between 2 and 6 mmHg in the healthy eye and to much higher, pathological levels in glaucoma (David, Zangwill, Briscoe, Dagan, Yagev, & Yassur, 1992; Liu et al., 1998; Phelps, Woolson, Kolker, & Becker, 1974).

Analysis of whole eye biometry shows that central corneal thickness (CCT), corneal curvature (1/R), anterior chamber depth (ACD), axial length (AL), and vitreous chamber depth (VCD) can all fluctuate during the day, and some of these changes are linked to IOP fluctuations (Chakraborty, Read, & Collins, 2011; Chakraborty, Read, & Collins, 2013; Harper, Boulton, Bennett, Marcyniuk, Jarvis-Evans, Tullo, & Ridgway, 1996; Kiely, Carney, & Smith, 1982; Read, Collins, & Iskander, 2008; Stone et al., 2004). Kiely et al. (1982) showed that the cornea becomes steeper during the day, but that this is not a consequence of changes in intraocular pressure. Some studies have reported links between IOP fluctuations and axial length, whereas others have not found any relationship (Chakraborty et al., 2013; Leydolt, Findl, & Drexler, 2008; Read et al., 2008; Stone et al., 2004). It is assumed, that increased IOP causes sclera loading, which increases the axial length of the eye (Pruett, 1988).

It appears that changes in eye biometry accompanying IOP fluctuations should significantly affect the quality of the vision. As a result of the diurnal variations in the axial length, which is shorter in the evening, progression toward a hyperopic state could be expected (Stone et al., 2004). However, the spherical equivalent refraction (about 0.4 D) indicates that a myopic shift occurs later in the day (Chakraborty et al., 2013; Schanzlin et al., 1986). Despite temporary fluctuations of the IOP and biometry of the eye, the quality of vision does not change. Spectacle-corrected and uncorrected visual acuities typically vary up to one Snellen line throughout the day, which, although clinically measurable, is not appreciated visually (Nizam et al., 1992; Schanzlin et al., 1986). Some studies have reported a positive association between IOP and myopia development (Quinn, Berlin, Young, Ziylan, & Stone, 1995). Conversely, eyes with myopic refractive error, a flatter cornea, and longer axial length are thought to be at higher risk of having open-angle glaucoma (Mitchell, Hourihan, Sandbach, & Wang, 1999; Wong, Klein, Klein, Knudtson, & Lee, 2003). The causal relationship between glaucoma and myopia is not clear.

It has been shown that shortening of the axial length occurs after IOP reduction, whether this is pharmacologically induced during trabeculectomy (Arranz-Marquez and Teus, 2004; Leydolt et al., 2008; Quaranta, Gandolfo, Turano, Rovida, Pizzolante, Musig, & Gandolfo, 2006) or is a result of drainage device surgery (Alvani, Pakravan, Esfandiari, Safi, Yaseri, & Pakravan, 2016; Cashwell & Martin, 1999; Francis, Wang, Lei, Du, Minckler, Green, & Roland, 2005; Kook, Kim, & Lee, 2001). After trabeculectomy, a significant shortening of the axial length of the eye is observed with a simultaneous change in the keratometric power (Alvani et al., 2016). A mechanically induced increase in IOP was reported to cause the eyeball to lengthen, with no effect on the thickness and position of the lens or ACD (Leydolt et al., 2008). Other studies have found that ACD decrease is caused by drugs that lower IOP without affecting visual acuity or changing the thickness of the lens (Gutiérrez-Ortiz, Teus, & Bolivar, 2006).

Corneal curvature has been reported to flatten in association with diurnal IOP changes, as a result of a Valsalva maneuver or inverted positions of measured subjects fluctuations (Chakraborty et al., 2011; Hjortdal, Böhm, Kohlhaas, Olsen, Lerche, Ehlers, & Draeger, 1996; Kiely et al., 1982; Thomas, Martinez, Nieves, & Applegate, 1991). These findings, however, are not definitive and other studies have not found any significant effects on corneal curvature (Asejczyk-Widlicka & Pierscionek, 2008; Feldman, Frucht-Pery, Weinreb, Chayet, Dreher, & Brown, 1989; Lam & Douthwaite, 1997; McMonnies & Boneham, 2007; Pierscionek, Asejczyk-Widlicka, & Schachar, 2007).

Significant changes in IOP, ocular pulse amplitude, and axial length have occurred in young, healthy adult subjects following ingestion of fluid (Read & Collins, 2010). Furthermore, CCT, ACD, and lens thickness have been shown to change. Although unlikely to be of clinical significance, these findings highlight the fact that hydration levels can influence ocular dimensions.

The effect of IOP on ocular biometrics is not clear, and a detailed analysis of biometric changes caused by IOP variability and its impact on image quality is difficult to conduct in vivo. It has been suggested that the optics of the eye undergo self-adjustment for dynamic changes in order for the image to maintain optimal quality (Asejczyk-Widlicka, Srodka, Kasprzak, & Iskander, 2004). Changes in IOP, particularly if these are rapid and large enough to affect the relative position of the ocular elements, could impact the quality of vision. These changes can potentially shift the corneal apex relative to the retina, and such a displacement, if sufficient to alter image quality, would have an effect on vision.

Given that in a healthy eye with all its concomitant dynamics (IOP daily fluctuations or ocular pulse and postural changes) the optics of the eye and the resulting vision are surprisingly robust, self-adjustment is entirely feasible to maintain sharp and stable imagery on the retina. How this occurs is not known. It could be explained by accommodation, for which the trigger is the blur of the retinal image (Sharmin & Vohnsen, 2019). This is only possible with negative defocusing if the light is focused behind the retina. However, Sharmin and Vohnsen (2019) showed that small fluctuations in the stimulus to accommodation (in the range of 0.25 D) do not induce an accommodative response, which is especially noticeable for larger pupil sizes. Sharmin and Vohnsen (2019) investigated the reaction of the lens to a variable stimulus up to 2.5 D, appearing at a frequency of 0.1 Hz. The findings of Sharmin and Vohnsen (2019) show that accommodation is not fast enough to exclude a consciously driven neural accommodation response. It is possible that the lack of change in accommodation could be compensated for by another mechanism. One plausible solution may be the self-adjusting effect proposed here.

Considerations of the self-adjusting effect could also explain the pseudoaccomodation with monofocal IOLs. In some cases, eyes that were corrected for distant vision with a monofocal IOL (nonaccommodative) also achieved a high level of near visual acuity with distance correction after cataract surgery (Leyland, Langan, Goolfee, Lee, & Bloom, 2002). Maybe the explanation is not only the activity of the muscles but also the effect of the self-adjustment.

Previously, self-adjustment was analyzed in the context of mechanical changes using numerical simulations such as finite element modeling (Asejczyk-Widlicka et al., 2004). A linear relationship between IOP and the axial length of the eye has been reported (Srodka & Kasprzak, 1997). Changes in IOP produced shifts of the corneal apex and focal point of the eye that corresponded to variations in the axial and focal lengths, respectively.

The purpose of this study was to investigate how self-adjustment may occur using an optical model of the eyeball and to test the requisite optical and biometric conditions. The study considered the biometric relationships between corneal curvature, anterior chamber depth, vitreous chamber, and axial and focal lengths during simulation of IOP changes.

## Methods

**Figure 1.**
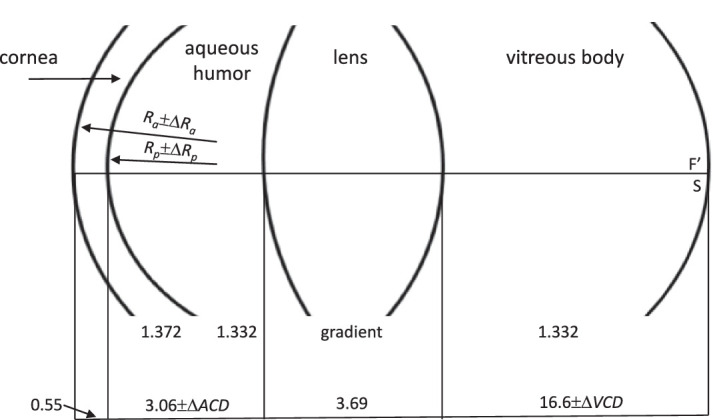
Parameters of Goncharov eye model for healthy 30-year old eye (Goncharov & Dainty, 2007).

OpticStudio 18.7 (Zemax, Seattle, WA) was used for the numerical calculations. Parameters of the eye were adopted from Goncharov eye model for an emmetropic eye with axial length of 23.9 mm and optical system power of 60.13 D (Figure 1) (Goncharov & Dainty, 2007). The Goncharov widefield schematic eye model with gradient-index lens includes four aspherical refractive surfaces, representing the cornea, lens, and aspherical retina (Table 1).

**Table 1.** Parameters of the Goncharov eye model for a healthy 30-year old eye (Goncharov & Dainty, 2007).

	Radius of curvature (mm)	Asphericity	Refractive index	Thickness (mm)
Cornea	7.76	–0.1	1.372	0.55
	6.52	–0.3		
Aqueous humor	—	—	1.332	3.06
Lens	11.51	–1.0	Gradient	3.69
	–7.67	0.96		
Vitreous	—	—	1.332	16.6
Retina	–12.0	0.5	—	—

Calculations were made for a pupil size of 3 mm and a monochromatic beam (λ = 589 nm). During simulation, corneal curvatures of the anterior (Δ*R*_a_) and posterior (Δ*R*_p_) surfaces and the position between the cornea and the lens—anterior chamber depth (Δ*ACD*)—were altered. Changes in lens position relative to the retina (Δ*VCD*) is dependent on Δ*ACD*, with the factor *k* (*k* ⋅ Δ*ACD*) representing the relative movements of the cornea and the lens with respect to the retina. Thicknesses of the cornea and lens are assumed to be constant; the calculations are for distance vision and do not take into account accommodation (Chakraborty et al., 2011). Most of the work explaining accommodation is based on blur as a stimulus. Cholewiak et al. (2018) showed that it is possible if defocus and chromatic aberration are turned on at the same time, in which case the accommodation can be adjusted so that the middle wavelengths (520 nm) are less blurred than the short (449 nm) and long (617 nm) wavelengths, with the short and long wavelengths being blurred a similar amount (Cholewiak et al., 2018).

**Figure 2.**
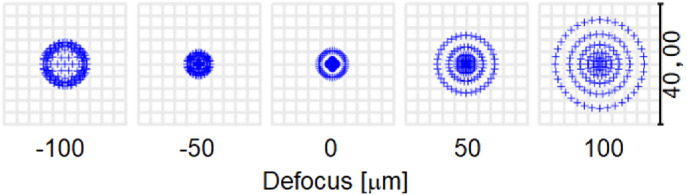
Spot diameters as criteria for image quality assessment.

In this study, the quality of vision was assessed by the analysis of minimum spot diameters formed on the retina. Spot diameter less than 5 µm with root mean square error less than 2 µm was obtained for each configuration (Figure 2). These values correspond with size of the cones in the macula and hence resolution of vision. The Strehl ratio was obtained at a level greater than 0.9. Sharmin and Vohnsen (2019) showed that the diameter of the focal spot size with defocus when normalized to a 2.5-µm center-to-center cone spacing decreases for different pupil diameters. Changes in the radii of corneal curvatures and the relative positions of the cornea, lens, and retina are required for maintenance of ocular image quality when the eye is subjected to small variations in IOP.

The value of parameter *k* was determined from changes in the ACD in the range from –0.5 to 0.5 mm (in the positive and negative direction) with incremental steps Δ*ACD* = 0.1 mm. This range of values of ±0.5 mm was based on previous studies and was greater than diurnal biometric changes (Alvani et al., 2016; Chakraborty et al., 2011; McMonnies & Boneham, 2007) to ensure that it covered all physiological norms and to determine the extent of linearity. Figure 1 shows the effect of diurnal changes in biometric parameters caused by daily IOP fluctuations on the defocus of the eye. The ranges of changes in biometric data selected from the literature were as follows: CCT, ±0.006 mm; ACD, ±0.05 mm; AL, ±0.032 mm; VCD, ±0.06 mm, and *R*_a_, ±0.4 mm (Alvani et al., 2016; Chakraborty et al., 2011; McMonnies & Boneham, 2007). Image quality indicated that biometric fluctuations using these ranges caused a defocus of less than 0.1 D, which is imperceptible for vision.

For each change in the Δ*ACD* value, we determined the changes in the radii of corneal curvature (Δ*R*_a_ and Δ*R*_p_) necessary to meet the requirements of self-adjustment. The ratio between the values of the radii of the anterior and posterior corneal surfaces was maintained as follows:

(1) $$\frac{R_{p}}{R_{a}}=\frac{R_{p}+\Delta R_{p}}{R_{a}+\Delta R_{a}}=0.824$$

Anterior chamber depth and vitreous body chamber thickness were altered in different proportions, as represented by parameter *k*. For every value of parameter *k*, the procedure was repeated to obtain the respective values of the ratio between changes in the Δ*R*_a_ and the anterior chamber depth, Δ*ACD*.

In addition to the method described above, three other versions of calculations were tested to obtain a combination of changes that would maintain image quality and that were physiologically plausible. The following combinations were taken into account, and respective relative changes in the geometrical parameters were assumed according to the following cases:

1. Corneal radii are constant (Δ*R*_a_ = 0 and Δ*R*_p_ = 0) and self-adjustment occurs by mutual changes in anterior chamber depth (Δ*ACD*) and length of the posterior chamber (Δ*VCD*). The result of this procedure is determination of parameter *k*.
2. Thickness of the vitreous body is constant (*k* = 0, so Δ*VCD* = 0), and the corneal radii are variable (in accordance with Equation 1). The anterior chamber depth is variable (Δ*ACD*). The result of this procedure is determination of the ratio Δ*R*_a_/Δ*ACD*.
3. Anterior chamber depth is constant (Δ*ACD* = 0) and the corneal radii are variable (according to Equation 1). The vitreous chamber depth is variable by 0.1 mm to a value of 0.5 mm (Δ*VCD*). The result of this procedure is determination of the ratio Δ*R*_a_/Δ*VCD*.

For the above calculations, a range ±0.25 D of refractive error (ΔΦ) was taken as tolerable and not clinically significant in terms of the visual acuity. This level of refractive error is caused by a shift of the focal position relative to the retina (∆*f*′) by about 0.1 mm (Alba-Bueno & Milan, 2011).

## Results

**Figure 3.**
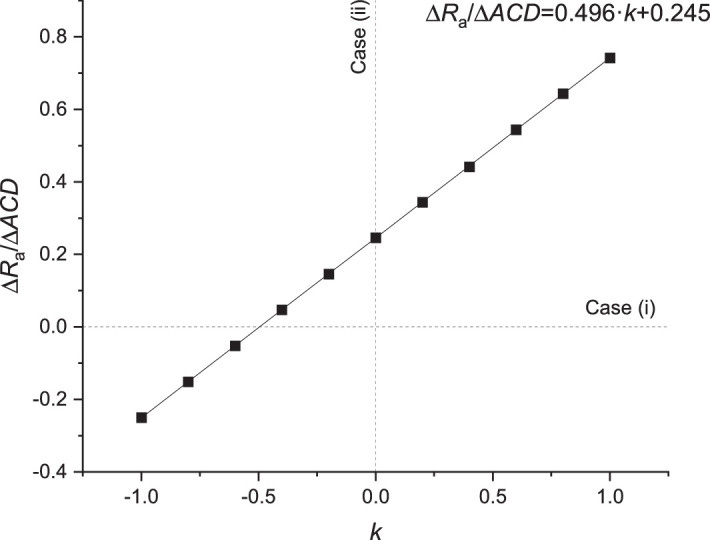
The relationship between ∆*R*_a_/∆*ACD* and parameter *k* for a constant ratio between radii of corneal curvatures. Dashed lines represent various combinations of the self-adjustment mechanism that were considered.

From analysis of the corneal shape change and position of the optical elements, the ratio Δ*R*_a_/Δ*ACD* was calculated for different values of *k*, and this relationship is presented in Figure 3. In this case, the radii of corneal curvatures, the anterior chamber depth, and vitreous chamber depth were changed. The relation between Δ*R*_a_/Δ*ACD* and parameter *k* is linear, with a high coefficient of determination (*R*^2^  ≈ 1) (Figure 3). On the basis of this parameter, changes in ACD, VCD, and corneal curvature that maintain self-adjustment can be deduced. There is one possible relationship between the changes in the values of these parameters that is needed to obtain self-adjustment. For a given value of *k*, a single value of Δ*R*_a_/Δ*ACD* will produce and maintain self-adjustment. Figure 3 shows the conditions required for self-adjustment in cases 1 and 2 (see Methods). In these cases, the condition for self-adjustment will be fulfilled when Δ*ACD*/Δ*VCD* = –0.487 for case 1 and Δ*R*_a_/ΔACD = 0.245 for case 2. Case 3 cannot be shown in Figure 3 because in this case Δ*ACD* is constant, and there is no given value of *k*.

**Figure 4.**
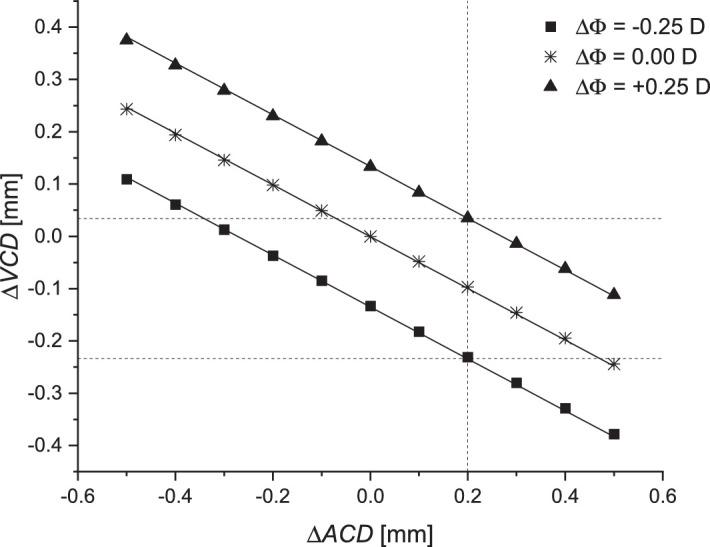
Correlation between change of the location of cornea (Δ*ACD*) and change of lens–retina distance (Δ*VCD*) with constant corneal radius; the correlation index as the *k* parameter in the range of refractive error of ±0.25 D (case 1).

When self-adjustment required to mitigate IOP fluctuations depends only on the relative positions of the cornea, lens, and retina with no change in the corneal radius (Δ*R*_a_ = 0 and Δ*R*_p_ = 0), the value of parameter *k* is equal –0.487 for ∆Φ = 0 D (Figure 4). This means that displacement of the lens is 49,6% that of the corneal displacement. For ∆Φ = 0 D and a change of Δ*ACD* in the range of ±0.5 mm, the axial length of the eye changes by 0.257 mm, and the optical power of the eye changes by 0.35D. These dependencies also remain linear over larger ranges of changes (exemplary up to ∆*ACD* = 1.5 mm). Nonlinearities in analyzed characteristics appear far beyond the range of physiological values of the considered variable biometric parameters of the eye.

Figure 4 indicates what is required to estimate the size of changes in optical parameters in order to maintain optical self-adjustment. If the anterior chamber depth changes by Δ*ACD* = 0.2 mm, then the vitreous chamber depth should change (Δ*VCD*) in the range of –0.231 to 0.035 mm from the initial value in order to maintain image quality. Assuming that self-adjustment is only associated with a change in the anterior chamber depth (Δ*VCD* does not change), the self-adjustment condition is fulfilled for Δ*ACD* in the range from –0.274 to 0.273 mm. Maintaining Δ*ACD* = 0 requires a change of Δ*VCD* ranging from –0.134 to 0.133 mm to maintain defocus at less than 0.1 mm.

**Figure 5.**
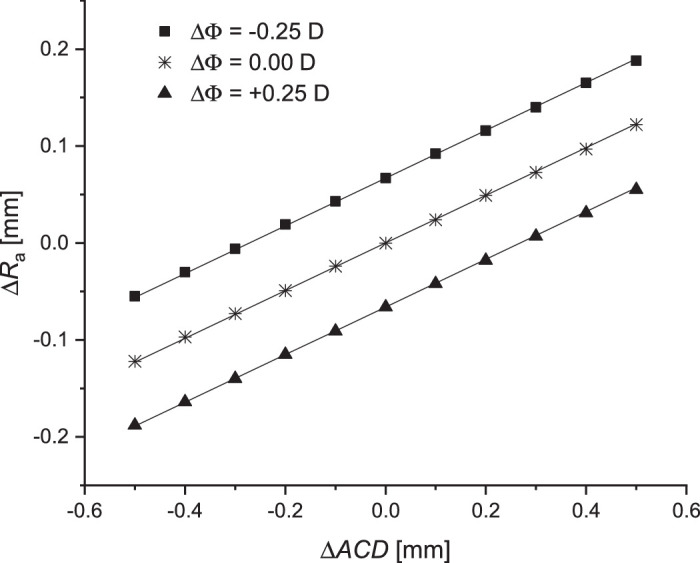
The relationship between ∆*R*_a_ and ∆*ACD* for ∆*VCD* = 0 and the constant ratio of radii of corneal curvatures (case 2).

When simulating the case described in point 1, with self-adjustment assumed to be the result of changes in the cornea radii and the depth of the anterior chamber, the ratios ∆*R*_a_/∆*ACD* and ∆*R*_p_/∆*VCD* were calculated. Simulations showed that, to maintain high-quality optical imagery, the corneal radii should change by 0.243 mm for the anterior surface and 0.205 mm for the posterior surface. Changes in the ACD and the corneal shape caused a change in optical power of 0.96 D for ∆*ACD* = –0.5 mm (*R*_a_ = 7.64 mm, *R*_p_ = 6.42 mm) and –0.93D for ∆*ACD* = +0.5 mm (*R*_a_ = 7.88 mm, *R*_p_ = 6.62 mm) for ∆Φ = 0 D. The relationship between ∆*R*_a_ and ∆*ACD* in the defocus range of ±0.25D is shown in Figure 5.

Assuming that self-adjustment is only associated with a change in the shape of the cornea (ACD and VCD do not change), the radii of curvature of anterior and posterior corneal surfaces should have the following values: *R*_a_ = 7.83 mm and *R*_p_ = 6.58 mm for a defocus of –0.25D, and *R*_a_ = 7.69 mm and *R*_p_ = 6.46 mm for a defocus of +0.25D. Such corneal shape changes are too large to be plausible physiologically, so such changes cannot be assumed to be involved in optical self-adjustment. The results of this calculations are presented in Figure 5 as points for Δ*ACD* = 0.

**Figure 6.**
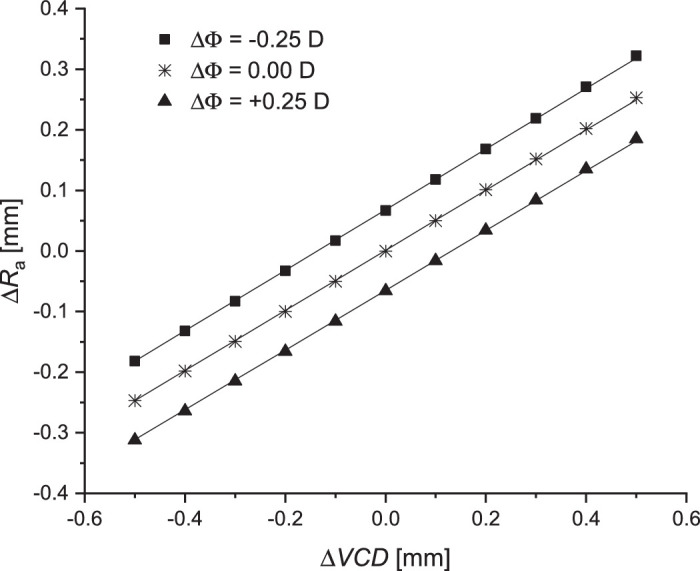
The dependence between ∆*R*_a_ and ∆*VCD* for the constant ratio of the anterior chamber depth (∆*ACD*) and between the radii of curvatures (case 3).

The literature reports changes in the axial length of the eye after surgical intervention to reduce IOP with no change in anterior chamber depth (David et al., 1992). Simulations based on this observation (as described in case 3) were conducted, and the results are shown in Figure 6. An increase in VCD by ∆*VCD* = –0.5 mm caused a change in ocular power of +1.26 D (*R*_a_ = 7.51 mm, *R*_p_ = 6.31 mm), and ∆*VCD* = +0.5 mm changed the power of the eye by –1.21 D (*R*_a_ = 8.01 mm, *R*_p_ = 6.73 mm); yet, the image on the retina remained in focus.

In summary, all of the mechanisms of self-adjustment are linked to changes in the optical system of the eye. Specific changes in the biometric parameters may vary in their contribution to preventing defocus. The values of biometric changes required to induce a shift of +0.5 D are shown in Table 2. A refractive power change of +0.5 D would not produce significant differences between the eyes and hence maintain binocular vision.

**Table 2.** Biometric changes required to induce a shift of ±0.5 D.

Case	–0.5 D	+0.5 D
1	∆*ACD* = –0.721 mm ∆*VCD* = 0.351 mm	∆*ACD* = 0.719 mm ∆*VCD* = –0.350 mm
2	∆*ACD* = 0.259 mm ∆*R*_a_ = 0.063 mm	∆*ACD* = –0.269 mm ∆*R*_a_ = –0.066 mm
3	∆*VCD* = 0.210 mm ∆*R*_a_ = 0.106 mm	∆*VCD* = –0.196 mm ∆*R*_a_ = –0.097 mm

## Discussion

The quality of the retinal image depends on the integrity of the optical system of the eye and dynamics of the eye; such fluctuations in optical biometry are linked with the material properties of the eyeball and its components. The aim of this study was to identify biometric parameters of the eye that may significantly affect and adjust for maintenance of retinal image quality. IOP also requires the globe to have sufficient flexibility to tolerate and adapt to fluctuations. Such fluctuations may cause the corneal apex to be displaced relative to the retina, as well as changes in the axial radius of corneal curvature (Kowalska, Kasprzak, Iskander, Danielewska, & Mas, 2011).

The first linear numerical model of the eye insensitive to image sharpness distortions caused by intraocular pressure changes was described by Asejczyk-Widlicka et al. (2004), who focused on the selection of trigonometric functions to model the relationships between the cornea, sclera, and limbus and self-adjustment in the optical system of the eye. This work reported the extent to which the biometric parameters of the eye would have to change to maintain image quality, as well as changes that would be physiologically feasible.

Mutual compensation of the defocus effect was analyzed by changing corneal radii (Δ*R*_a_ and Δ*R*_p_) and depth of anterior chamber and vitreous body (Δ*ACD* and Δ*VCD*) in a range allowing for ±0.25 D of refractive error. Assuming that Δ*VCD* changes depending on how Δ*ACD* and Δ*R*_2_ change in proportion to Δ*R*_a_, we determined the linear dependence between Δ*R*/Δ*ACD* and parameter *k*. From this relationship, it is possible to determine how the above biometric parameters must change in order to achieve optical self-adjustment.

Based on these findings, we proposed a numerical simulation in which it was assumed that the corneal shape would not be changed (Δ*R*_a_ = 0 and Δ*R*_p_ = 0) and that changes between Δ*ACD* and Δ*VCD* would be linear (Figure 4). However, if the distance between the cornea and the lens increases, then we would expect the distance between the lens and the retina (Δ*VCD*) to decrease by about half of Δ*ACD* (parameter *k* = –0.487 for no defocus) to ensure self-adjustment in the optical system of the eye. This result is supported by Leydolt et al. (2008). Using Silver's formula (Silver & Geyer, 2000) for the pressure–volume relation based on previous measurements of ocular rigidity made on living human eyes, the calculated volume reduction as a result of the reduced axial length (6 mL) and the effect of backward movement of the posterior lens pole and, therefore, anterior vitreous surface (10 mL) should lead to a total loss of vitreous volume of 16 mL, on average.

Quigley, Friedman, and Congdon (2003) reported that an increase of pressure behind the vitreous (e.g., choroidal swelling or scleral compression) increased the absolute pressure difference within the eye more in the posterior segment than in the anterior segment. As a consequence, fluid passes from the vitreous and into the posterior chamber of the anterior segment, where it exits through the trabecular meshwork and uveoscleral outflow paths. Therefore, simultaneous deepening of ACD should be observed, as shown in our study.

For a constant value of ∆*VCD*, a linear relationship was found between ∆*R*_a_ and ∆*ACD*, such that the ratio ∆*R*_a_/∆*ACD* should equal 0.243 to maintain retinal image quality. Similar results were obtained by Kasprzak (1997). A linear relationship was also obtained for the relationship between ∆*R*_a_ and ∆*VCD* for a constant ratio of anterior chamber depth, ∆*ACD* (Figure 6). If self-adjustment is associated only with a change in the depth of the anterior chamber, then self-adjustment occurs for Δ*ACD* ranging from –0.274 to 0.272 mm. Similarly, if only Δ*VCD* is variable, that value ranges between –0.134 mm and 0.133 mm to maintain image quality.

The self-adjustment mechanism must be experimentally tested in order to be verified. Two other models were also tested (Atchison, 2006; Liou & Brennan, 1997) and found to be within 0.1% of the dioptric power found with the Goncharov model. It is possible that the response will vary with individuals and may be linked to (a) thickness of the cornea and sclera, axial length, and anterior and vitreal chamber depths; (b) optics of the eye and refractive status; (c) age and degree of ocular and systemic health; and (d) biomechanical properties. Information about how the eye maintains clarity of vision may provide essential understanding of the interplay between optical and biomechanical properties and would be of great interest to optical designers, computer vision specialists, and ultimately for researchers who are striving to develop a bionic eye. A fundamental aspect of self-adjustment is the very nature of the process: Is this passive or active? Does it rely on feedback from the retina and higher visual pathways, or are the different ocular components finely tuned to changes in related components? Such information could lead to adjustment via learned behavior, genetics, or epigenetic programming so that when one component is stretched or moved another reacts to meet the requirements of clear vision. Alternatively, the process could be largely passive, given that all of the components of the eye are physically connected, either directly by being in close contact or indirectly by being in contact with similar bodies (the lens and cornea are both in contact with the aqueous humor). These questions and unknowns require further, detailed experimentation.

## Conclusions

The eyeball is subjected to small daily variations in IOP, and these processes exert forces on the outer surface of the eyeball and on the cornea, causing changes in axial length and corneal shape. These changes act in synchrony in order to preserve image quality on the retina. Doing so requires a balance between the rheological properties of the cornea and sclera, as well as a stabilizing feature that can maintain the corneal shape by adjusting it in response to IOP changes. Optical modeling can indicate where changes in ocular biometry are likely to occur. Experimental work is necessary to test and investigate the nature of such a mechanism.
